# Established and emerging non-cellular therapies in inherited bone marrow failure syndromes

**DOI:** 10.3389/fimmu.2026.1773574

**Published:** 2026-03-04

**Authors:** Szymon Janczar, Bartosz Urbański, Marek Ussowicz, Wojciech Mlynarski

**Affiliations:** 1 Department of Pediatrics, Oncology and Hematology, Medical University of Lodz, Lodz, Poland; 2 Department of Paediatric Bone Marrow Transplantation, Oncology and Hematology, Wroclaw Medical University, Wroclaw, Poland

**Keywords:** cytopenia, drug repurposing, inherited bone marrow failure syndrome, secondary cancer, steroids

## Abstract

Inherited bone marrow failure syndromes (IBMFs) are a molecularly heterogeneous group of genetically-determined syndromes with a wide spectrum of clinical abnormalities and risk of hematopoietic neoplasia and reduced life expectancy. The prevalence is not well established but is growing due to the advent of genetic diagnostics. Allogeneic hematopoietic stem cell transplantation (HSCT) remains the only curative modality in eligible IBMF patients but the indications are individualized due to significant transplant-related morbidity, including secondary cancer. HSCT indications and practices are also impacted by the gradual introduction of gene therapies for IBMFs, but that remains developmental at the moment. Medical non-transplant therapies have played an important role in several IBMFs for decades. Their impact vary from nearly curative treatment in Diamond–Blackfan Anemia (DBA), through supportive interventions, to experimental treatment aimed at impacting the natural course of the disease. This may be subject to change, because of the description of new syndromes or new pathomechanisms of the established syndromes. While their impact might be diminishing due the progress in gene therapy, there might be some new clinically useful interventions due to new medical technologies and the progress in drug-repurposing. The medical therapies can be used as a mainstay of therapy, bridge therapy to transplantation, peri- or post-transplantation aiming at amelioration of the hematological and non-hematological phenotype, improved growth and development, diminishing the risk of myeloid neoplasm or other cancer. Disease- and therapy-specific risks of these interventions must be actively addressed in patients. We present state-of-the-art and developmental applications of non-cellular therapies in IBMFs.

## 1 Introduction

### 1.1 Prevalence, symptoms and natural history

Inherited bone marrow failure syndromes (IBMFs) are a molecularly heterogeneous group of genetically-determined syndromes with severe clinical behavior, dependent primarily on deficient production of blood cells in the bone marrow, with risk of clonal progression to hematopoietic neoplasia. They are associated with very significant morbidity, ranging from life-threatening infections to extrahematopoietic signs, but the major cause of mortality is blood cancer, in particular myeloid neoplasms. Other cancers are also observed, especially in some IBMFs (1–7).

While there are some surprising cases with relatively few disease symptoms, IBMFs belong to most debilitating diseases of childhood, associated with low quality of life and significant mortality (3, 7–9). The specific problems include cytopenias and their complications, especially severe infections and transfusion-dependence. There are also frequent features of immunodeficiency and immune dysregulation exceeding that expected purely due to low leukocyte count such as occasional features of combined immunodeficiency and atypical infections in patients with for example GATA2 or SAMD9/L defects. There are syndrome-specific, extrahematopoietic manifestations including growth failure, neurological deterioration, skin abnormalities, gastrointestinal and pancreatic disorders, lung disease, skeletal abnormalities and others. Life expectancy is much shorter than in general population (1, 2, 4, 6, 7, 10).

The prevalence of IBMFs is not well known, as the IBMFs field and rate of diagnosis is much changing as a consequence of rapid improvement of availability of modern genetic diagnostics and awareness of the disorders. A new IBMF syndrome is described every few years. Some authors estimate the cumulative prevalence at approximately 65 per million live births, but under-diagnosis is likely. The most prevalent IBMFs are believed to be Fanconi Anemia (FA) and Diamond-Blackfan Anemia (DBA), roughly at 1: 150–000 births (3–5, 11, 12). On the other hand, there is a steep rise in the number of patients with newly described SAMD9/L deficiency syndrome, and the cumulative IBMFs prevalence might be much higher than initially thought (13, 14).

### 1.2 Etiology and pathogenesis

**Figure 1:**
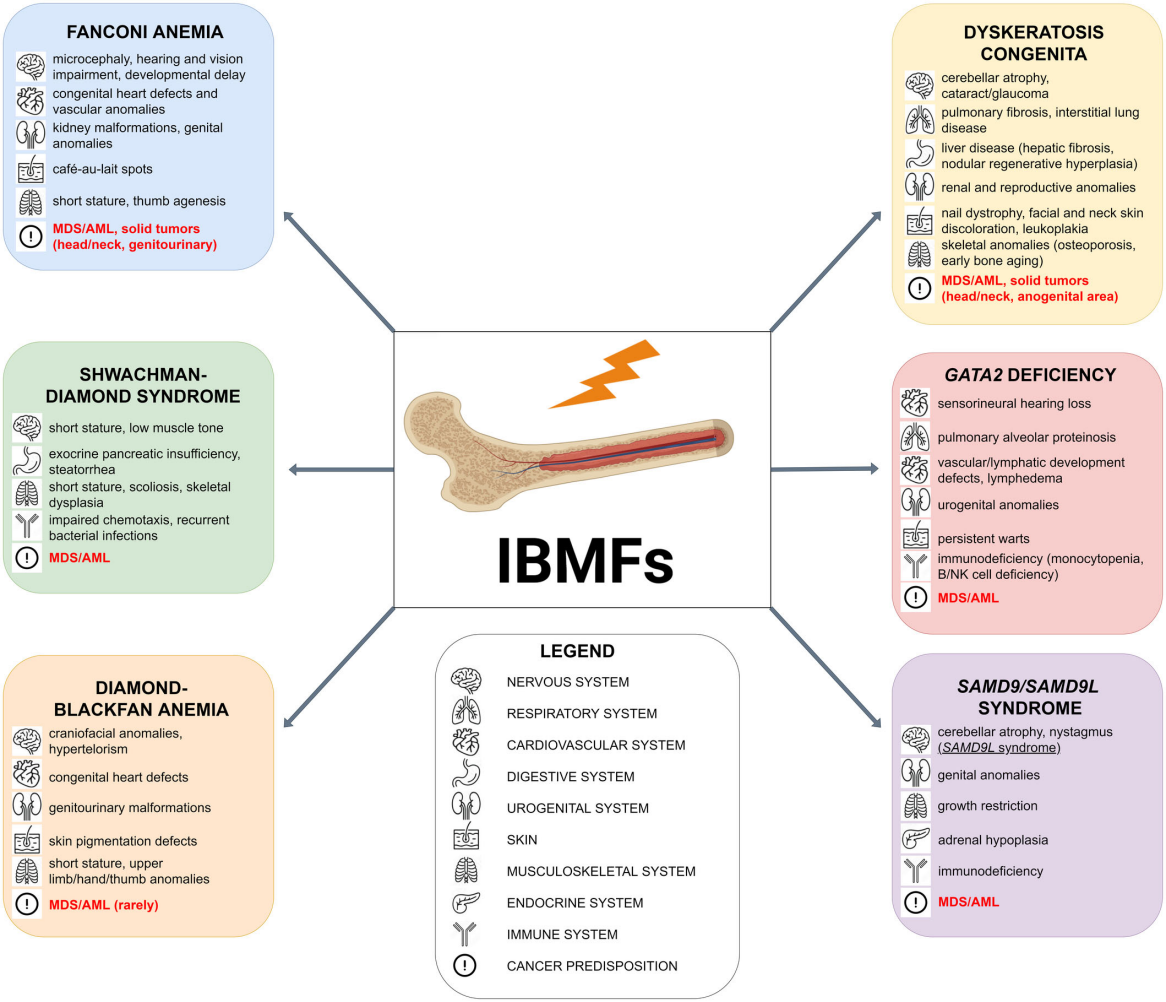
Overview of extrahematopoietic manifestations of the most common inherited bone marrow failures (IBMFs) and their associated risk of malignant transformation.

With very few exceptions IBMFs are monogenic disorders due to pathogenic variants in genes with potential impact on hematopoiesis (**Table 1**). In a small proportion of cases there is a strong suspicion of an inherited syndrome but no clear molecular diagnosis. The disrupted molecular pathways are widely varied and include transcriptional regulation of differentiation, deficient growth factor signaling, telomere maintenance, ribosome biogenesis, RNA processing, DNA repair, and other phenomena. Some of these have a degree of specificity for one hematopoietic lineage (such as erythropoiesis failure in DBA or agranulocytosis in severe congenital neutropenia <SCN>), other cause multilineage cytopenia/dysplasia. There are syndromes with minimal symptoms beyond hematopoietic syndrome (such as some forms of SCN or inherited thrombocytopenia) or severe abnormalities in multiple systems (telomere-biology disorders/dyskeratosis congenital <TBD/DC> or Shwachman-Diamond syndrome <SDS>) (10–15), **Figure 1**.

**Table 1:** Overview of inherited bone marrow failure syndromes, associated genes, and non-cellular treatment options with rationale.

Disease	Involved genes	Medical agent/intervention [clinical status: standard in bold *developmental* in italics]	Disease-relevant mechanism of action
DBA	RPS7, RPS10, RPS15A, RPS17, RPS19, RPS20, RPS24, RPS26, RPS27, RPS28, RPS29, RPL4, RPL5, RPL8, RPL9, RPL11, RPL15, RPL17, RPL18, RPL26, RPL27, RPL31, RPL35, RPL35A, TSR2, HEATR3, GATA1	Glucocorticosteroids	Gene expression regulation correcting compromised DBA-erythropoiesis at several levels
		*Leucine*	Improvement of translational efficiency through activation of the eukaryotic translation initiation factor 4E-BP1 and mTOR signaling, up-regulation of ribosome synthesis, enhanced erythropoiesis
		*TPO-RA*	JAK/STAT activation, improved expansion and maintenance of hematopoietic stem cells
		Chelation therapy (deferoxamine, deferasirox)	binding free iron in the bloodstream and enhancing its elimination in the urine (deferoxamine) or feces (deferasirox)
SDS	SBDS, EFL1, DNAJC21, SRP54	Pancreatic enzymes	Correction of exocrine pancreatic dysfunction
		Fat-soluble vitamins	Correction of fat-soluble vitamin deficiency
		G-CSF	Stimulation of neutrophil maturation and survival
		*Ataluren*	Misreading the premature termination signal to produce a full-length polypeptide in non-sense mutated monogenic syndromes
FA	FANCA, FANCB, FANCC, FANCD2, BRCA2, FANCE, FANCF, XRCC9, FANCI, BRIP1, FANCL, FANCM, PALB2, RAD51C, SLX4, ERCC4, RAD51, BRCA1, UBE2T, XRCC2, MAD2L2, RFWD3	Androgens	Gene expression regulation correcting compromised DC-hematopoiesis at several levels
		*Metformin*	Activation of AMPK, scavenging reactive aldehydes
		*Quercetin*	Free radicals scavenger, inhibition of lipid peroxidation
		*TPO-RA*	JAK/STAT activation, improved expansion and maintenance of hematopoietic stem cells
TBD/DC	TERT, TERC, DKC1, TINF2, RTEL1, ACD, NOP10, NHP2, WRAP53, PARN, RPA1, TERF2IP, CTC1, NAF1, POT1	Androgens	Gene expression regulation correcting compromised DC-hematopoiesis at several levels, telomere elongation
SCN/CyN	ELANE, CLPB, HAX1, CSF3R, JAGN1, TAZ, SRP72, VPS13B, VPS45, WASP	G-CSF	Stimulation of neutrophil maturation and survival
		Mavorixafor	CXCR4 antagonist
GSD type 1b	SLC37A4	Empagliflozin	Reduction of myelotoxic 1,5-anhydroglucitol-6-phosphate (1,5AG6P) accumulation
G6PC3-deficiency	G6PC3	Empagliflozin	Reduction of myelotoxic 1,5-anhydroglucitol-6-phosphate (1,5AG6P) accumulation
WHIM	CXCR4	Plerixafor, Mavorixafor	CXCR4 antagonist
Barth Syndrome	TAZ	Elamipretide	Binding to cardiolipin on the inner mitochondrial membrane to correct mitochondrial structure, electron transport chain function and ATP production (no data on hematological response).

DBA -Diamond-Blackfan Anemia, SDS -Shwachman-Diamond Syndrome, FA-Fanconi Anemia, TBD/DC -Telomere-Biology diseases/Dyskeratosis congenita, SCN -severe congenital neutropenia, CyN -cyclic neutropenia, GSD type 1b -glycogen storage disease type 1b, G6PC3-deficiency - glucose-6-phosphatase catalytic subunit 3 deficiency, WHIM –WHIM (warts, hypogammaglobulinemia, infections, and myelokathexis) syndrome.

### 1.3 Management

For several decades allogeneic hematopoietic stem cell transplantation (HSCT) has been and largely remains the only curative modality in inherited bone marrow failure syndrome. The indications are individualized and remain disease and disease-phase specific because of significant transplant-related morbidity and difficulty to gather high-level medical evidence of appropriate quality and volume in this heterogeneous group of rare disorders. HSCT is a high-risk procedure, and the risk might be aggravated by severe complications of myeloablative conditioning in some syndromes. While HSCT is currently the only definite approach, there is a significant rate of transplant-related mortality, and in some patients quality of life post HSCT might be very low due to for example chronic Graft-versus-Host Disease (GvHD) (8, 9, 16). There are also IBMFs-specific hazards that include in particular the increased risk of secondary solid cancer. Because of these pre- and post-transplant considerations and lack of high-level evidence, there is a lack of stringent criteria for pre-emptive HSCT prior to myeloid neoplasia and apart from disease-guided data the practice varies center-to-center and patient-to-patient. Also some patients might be not eligible for transplant or lacking a suitable donor (3, 6–9, 11, 12, 16, 17). This area and HSCT indications and practices are also impacted by the gradual introduction of gene therapies for IBMFs, but that remains developmental at the moment and have low impact on general management.

Medical non-transplant therapies have played an important role in several IBMFs from the early stages of the development of this area of hematology. Their impact varies from nearly curative treatment in Diamond–Blackfan Anemia (DBA), through supportive measure to control complications, to experimental treatment aimed at impacting the natural course of the disease. Accordingly, their part is fundamental in some of the disorders and quite minimal in others (3, 12, 18, 19). The role of medical non-cellular therapies in IBMFs may be subject to change, because of the discovery of new syndromes or new pathomechanisms of the established syndromes, potentially accumulating data from a so far limited number of available clinical trials and the emergence of new technologies in medicine. In general their impact might be diminishing due the progress in gene therapy, on the other hand there might be some new clinically useful interventions due to the progress in drug-repurposing and approaches such as targeted degraders (1–3, 7, 11, 12, 14, 18, 20).

The medical therapies can be used as a mainstay of therapy, bridge therapy to transplantation, peri- or post-transplantation and may, at least theoretically, have varied aims. The potential aims include: amelioration of the hematological phenotype, improvement of other organ function, improved growth and development, diminishing the risk of myeloid neoplasm or other cancer, control of infections or autoimmune manifestations (3, 11, 12, 20).

Following the introduction of the biology/mechanisms of the major classes of relevant agents the non-cellular therapies will be presented here with focus on clinical use in a disease-specific manner, and that divided into well-established and emerging/experimental approaches, this is also summarized in **Table 1**. This topic was reviewed in 2017 by Calado et al, but the knowledge expanded since that time (3).

## 2 Overview of the of major classes of medical therapies and their mechanisms of action in IBMFs

### 2.1 Glucocorticosteroids

Glucocorticosteroids are among the best known and most frequently used drugs in medicine and their biology and pharmacology are well described in basic medical textbooks and thus this is only very briefly described here. In a snapshot, glucocorticosteroids bind to the glucocorticoid receptor, resulting in nuclear translocation of the complex, binding DNA at glucocorticoid response elements and activating gene transcription (21). On the other hand, why glucocorticosteroids are effective in DBA, despite over 60 years of clinical experience, is much less obvious and the knowledge is expanding parallel to the expanding science of DBA. It is generally believed that glucocorticoids ‘unblock’ erythropoiesis at the erythroid progenitor level, the precise description and of the process and mechanisms remains elusive because of varied systems and markers used in relevant studies. The major cellular and molecular abnormalities in DBA include translational dysfunction, increased Inflammatory signaling pathway, unbalanced globin/heme synthesis, dysregulated autophagy, p53 activation and cell cycle arrest, and according to various reports all this pathways are to some extent corrected by glucocorticosteroids, but the data on precise mechanisms remains inconclusive. This complex pleiotropic mode is also surprising and difficult to apply taking into account the wide genetic/molecular and clinical heterogeneity of DBA. Still, the response to glucocorticoids and its perseverance is not universal and better understanding of the key erythropoiesis corrective mechanism may boost clinical practice (3, 12, 22–24). The practical considerations of glucocorticoid use in DBA are included below and are extensively presented and discussed elsewhere in recent recommendations (12, 23).

### 2.2 Androgens

Androgens have been used for several decades in IBMFs, mainly telomeropathies and also FA due to their beneficial effects on erythrogenesis and telomere regulation. The potential role of anabolic steroids in IBMFs was first based on the observation, early in the 20th century, of some cases of remission in young boys with aplastic anemia/marrow failure at the onset of puberty (18, 20, 25–28). They are used with varied success to treat patients who are not HSCT candidates or bridge those awaiting HSCT. Similarly to glucocorticosteroids, anabolic steroids act mainly through the interaction with androgen receptors in a DNA binding-dependent manner to regulate gene transcription. The effects on hematopoiesis in the setting of BMF are postulated to be related through several different pathways. The mechanisms include increased production of erythropoietin and up-regulation of erythropoietin receptor expression on erythroid progenitors, hepcidin inhibition resulting in increased iron mobilization, telomere elongation in hematopoietic stem cells (HSC) via binding to the estrogen response elements (ERE) present in the TERT gene promoter and modulation of bone marrow microenvironment. The evidence for telomere elongation following danazol treatment in dyskeratosis congenita is well documented (29). These affected pathways remain not well defined and are believed to cumulatively translate into improved cell survival and proliferation, and clinically beneficial effects on immune cell populations differentiation. There is a pharmacological and biological heterogeneity of androgens, with oxymetholone and danazol most frequently described in IBMFs (3, 18, 19, 25, 26, 30–33). The side effects are well documented and include virilization, acne, enlargement of penis or clitoris, premature closure of epiphyses and resultant short stature, behavioral changes such, cholestasis, hepatic tumors and hypertension. Danazol appears to have relatively favorable toxicity profile (20, 28). Disease-specific considerations are presented below.

### 2.3 Thrombopoietin-receptor agonists

The two best-known thrombopoietin receptor agonists (TPO-RA), eltrombopag and romiplostim, were licensed FDA for treatment of immune thrombocytopenia (ITP) in 2008. Since that time their use is steeply increasing and they are now widely used by hematologists around the world in ITP, but also in severe aplastic anemia, hepatitis C patients undergoing treatment with interferon-ribavirin and developmentally in post-chemotherapy thrombocytopenia, inherited thrombocytopenia, graft failure post hematopoietic stem cell transplantation and other indications. Romiplostim and eltrombopag both bind to the thrombopoietin (TPO) receptor, resulting conformational change in the TPO receptor, activation of the JAK/STAT pathway, and improving megakaryocyte progenitor proliferation as well as expansion and maintenance of hematopoietic stem cells (HSC). There are some differences between the two agents related to their binding sites in the TPO receptor and this extensively reviewed and discussed elsewhere (34–36). There are also reports suggesting that eltrombopag promotes C-NHEJ DNA repair in hematopoietic stem cells and this enhanced DNA repair may result in enhanced HSC genome stability with potential applications in FA (37–39). The applications of TPO-R in IBMFs are rather narrow and developmental, and are described in disease-sections below.

### 2.4 G-CSF

Granulocyte colony stimulating factor (G-CSF) is a glycoprotein initially discovered due to its ability to stimulate proliferation of bone marrow neutrophil progenitors *in vitro*. G-CSF is encoded by the CSF3 gene and can be produced by different tissues. G-CSF binds to a single receptor (G-CSFR; CSFR3) on the surface of target cells and it activates signaling associated with proliferation, differentiation, survival and other features of granulocytes. G-CSF supports the division and differentiation of myeloid precursors and promotes neutrophil maturation leading to increased neutrophil counts in peripheral blood. G-CSF is routinely used to ameliorate of chemotherapy-associated neutropenia (40).

Phase I–III clinical trials demonstrated that G-CSF increases neutrophil counts in SCN and some other bone marrow failures. G-CSF use significantly increased rates of survival of children with SCN and is in clinical use since 1980s (41–43). Disease-specific applications are presented below.

### 2.5 CXCR4 antagonists

WHIM syndrome (warts, hypogammaglobulinemia, infections, and myelokathexis) is a rare severe immunodeficiency caused by gain-of-function mutations in CXC chemokine receptor 4 (CXCR4). Multiple immune cellular compartments are affected in WHIM syndrome leading to defects in specific immunity, lymphopenia, neutropenia, and recurrent and specific (e.g. HPV-related warts) infections. Neutropenia is related to myelokathexis, which is a retention of neutrophils in the bone marrow caused by hyperactivity of CXCR4 and down-stream signaling effects. CXCR4 antagonist plerixafor, mainly licensed and used for hematopoietic stem cell mobilization to peripheral blood in transplantology, was demonstrated to correct cytopenia, reduce infections, including HPV, and improve quality of life of WHIM-patients (44–48). This is a successful example of mechanism-driven drug repurposing. Importantly, while plerixafor requires subcutaneous injections, another CXCR4 antagonist effective in WHIM, mavorixafor is administered orally (49). This is presented in a disease-specific manner below.

### 2.6 Quercetin

Studies in FA-animal and human models indicated that increased levels of reactive oxygen species (ROS) and high vulnerability of hematopoietic progenitors to ROS play a significant role in the pathogenesis of bone marrow failure in FA. Quercetin is plant-derived flavonoid with antioxidant properties. Its mechanisms include directly scavenging free radicals, catalysis of electron transport in the xanthine/xanthine-oxidase pathway, chelating metal ions and inhibiting lipid peroxidation (50). Quercetin was shown to reduce spontaneous and diepoxybutane (DEB)-, formaldehyde- and acetaldehyde-induced cell cycle arrest in cells from patients with FA and was postulated to have a potential to alleviate bone marrow failure and solid cancer risk, justifying entry into low-phase clinical trials (3).

### 2.7 Ataluren

Ataluren belongs to a class of Translational readthrough-inducing drugs (TRIDs) that have been designed to suppress nonsense mutations in inherited monogenic disorders. TRIDs misread the premature termination signal by inserting a near-cognate amino acid in the place of the premature termination codon to continue translation and produce a full-length polypeptide This group of agents is known for relatively long but recently received renewed interest. Phase I, II and III clinical trials, including nonsense mutation mediated Cystic Fibrosis (CF) and nonsense mutation mediated Duchenne muscular dystrophy (DMD) individuals, demonstrated the long-term safety, changes in the expression of full-length target proteins and benefits relative to placebo of ataluren. Ataluren (PTC Therapeutics) was conditionally approved for use in Duchenne muscular dystrophy (DMD) in Europe in 2014 (51). Currently, the marketing authorization in Europe has been withdrawn (on 28 March 2025), following the opinion issued by EMA, which recommended to not renew the authorization because the effectiveness of the medicine has not been confirmed. The FDA is continuing the review of ataluren therapy specifically for individuals with nonsense mutation Duchenne (DMD). These regulatory situation may negatively impact potential use in IBMFs. Preclinical data in non-sense mutation Shwachaman-Diamond syndrome models demonstrated SBDS protein re-expression in different cellular linages following ataluren exposure. This was associated with improved ribosome biogenesis, reduced p53 levels and improved myeloid differentiation in SBDS cells (52–54). There is also single-report of ataluren effects in an FA model (55).

### 2.8 Metformin

Metformin, a guanidine derivative, is a widely used oral antidiabetic drug with well-defined safety profile. There have been many attempts of metformin repurposing is several diseases. There are multiple cellular consequences of metformin administration but the antidiabetic effect is likely mainly related to the induction of activation of adenosine 5′-monophosphate–activated protein kinase (AMPK). Metformin was tested in preclinical FA models and tested clinically in the FA setting based on the potentially beneficial effects of the activation of AMPK on hematopoiesis and scavenging reactive aldehydes that are toxic to bone marrow cells in several IBMFs (3, 56–58).

### 2.9 SGLT2 inhibitors

Neutropenia in GSD type 1b and SCN-4 were demonstrated to be the consequence of 1,5-anhydroglucitol-6-phosphate (1,5AG6P) accumulation in granulocytes caused by the loss of G6PT1 or G6PC3 that normally collaborate to destroy this metabolite. Toxic concentrations of 1,5AG6P inhibit hexokinase activity, lead to poor glucose utilization and eventually cell death in both disorders.

The realization of the neutrophil loss mechanism in GSD type 1b and G6PC3-deficiency, motivated preclinical attempts at 1,5AG6P reduction, with an inhibitor of renal glucose transporter Sodium-glucose co-transporter-2 (SGLT2) yielding spectacular hematological recovery effect in murine models. Further, this approach was successfully attempted in patients with GSD type 1b and G6PC3-deficienciency using off-label empagliflozin. Re-purposing of this antidiabetic drug is an elegant example of mechanism-driven medical intervention (42, 59, 60).

### 2.10 Poly(A) polymerase PAPD5 inhibitors

Restoring or enhancing activity of telomerase, which is a ribonucleoprotein (RNP) enzyme that adds telomere repeat sequences to the 3’ end of telomeres, in cells of patients with telomere-biology disorders has been long considered a potential therapeutic intervention in TBD to achieve telomere elongation (61, 62). One of the approaches studied in preclinical models over the last decade was prevention of human telomerase RNA component (hTR) RNA decay, which is mediated by PAPD5 oligoadenylation executed by exonuclease EXOSC10 (PAPD5h adds 3’ oligo(A) tails to promote substrate recognition by EXOSC10) (63, 64). It was shown that using human embryonic stem cells (hESCs) with a dyskerin mutation (DKC1_A353V) and defective telomere maintenance that reduction of PAPD5 levels led to improvement in telomerase activity, telomere elongation and features of improved hematopoietic potential (65, 66).

## 3 Established and emerging non-cellular interventions in IBMFs

### 3.1 General measures (infection control, immunosuppression and anti-cytokine drugs)

#### 3.1.1 Antimicrobial agents

Appropriate control of infectious and inflammatory episodes limits the extent of organ complications and may potentially help avoid post-infectious deterioration of hematopoietic parameters (60). Occasionally, and in correlation with a degree of immunodeficiency, antibacterial, antifungal (including Pneumocystis jiroveci) or antiviral prophylaxis is used, based on individual clinical profile and history. This might be relatively frequently required in for example GATA2 deficiency, especially with failing hematopoiesis (67, 68).

#### 3.1.2 Immunoglobulins

Some of the IBMFs have the clinical picture or overlap with primary immunodeficiency (69). Somewhat surprisingly, telomere-biology disorders may present with CVID (common variable immunodeficiency) phenotype (70). Immunoglobulin substitution is warranted as per clinical and laboratory indications in particular in some IBMFs such as GATA2 deficiency, SMAD9/L deficiency or during prolonged immune reconstitution post HSCT (67, 69, 71).

#### 3.1.3 Immunosuppression and anti-cytokine drugs

Some IBMF patients may display autoimmune and autoinflammatory phenomena and based on rare indications immunosuppression or ‘biologic’ (anticytokine) drugs are administered as indicated by standard treatment in a recognized particular autoimmune/autoinflammatory disease. HLH-like episodes also require appropriate medical treatment. We refer the readers to a detailed presentation of the topic in our previous publication (60). In some IBMFs there is evidence for a general role of inflammatory signals in the pathogenesis and phenotype also increased inflammatory signaling is believed to be increased in myelodysplastic syndrome/neoplasm (60, 69, 72–76). Despite that but the use of anti-cytokines in IBMFs setting is scarce and, in particular, etanercept failed to elicit hematological responses in FA (72).

### 3.2 Diamond-Blackfan anemia

#### 3.2.1 Established interventions

These are comprehensively presented and discussed in a recent comprehensive position paper and thus is presented minimally here (23). These include oral steroids and treatment of iron overload (chelation therapy).

##### 3.2.1.1 Glucocorticosteroids

Glucocorticosteroids have been successfully used in management of DBA patients for nearly 8 decades. The principal therapy aim is to enable adequate growth, development and quality of life in children and adults, and maintaining hemoglobin concentration at a level of at least 9–10 g/dL with minimal steroid dose is considered as the target. Steroids can be started in transfusion-dependent patients, after 12 months of age, and preferably after administration of the first live vaccines (measles, mumps, rubella, varicella vaccines). The initial prednisone dose is 2 mg/kg daily in children or 80 mg/day in adults, once daily in the morning or divided in two equal doses. To assess for response, reticulocytes and hemoglobin are measured 10–14 days after starting steroids. If a significant reticulocytosis rise is observed and hemoglobin is at least stable, steroid tapering should start in 2–4 weeks, by 0.5 mg/kg per day approximately every 2 weeks until reaching 0.5 mg/kg per day. Late on, steroids should be tapered over months to find the lowest effective maintenance dose, that should not exceed 0.3 mg/kg per day (or 0.6 mg/kg every second day). The maintenance dose in adults should not exceed 15 mg/day. Further tapering strategies are not well established but some patients may require very low doses such as 0.05 mg/kg per day) to maintain response. If the response is lost, the dose should be to the previous effective dose. If the hemoglobin concentration cannot be maintained at or above 9 g/dL with 0.3 mg/kg per days, prednisone should be discontinued. If a patient does not respond to 2 mg/kg per day of prednisone within a month, the drug must be withdrawn. Lack of response to steroids is defined as failure to reach hemoglobin concentrations of at least 9 g/dL after 4 weeks of full-dose therapy, or a need for more than 0.3 mg/kg per day to keep a hemoglobin concentration of 9 g/dL. This statement recommends the use of equally potent oral prednisone or prednisolone (23).

##### 3.2.1.2 Chelation therapy

This is again described in full detail in recent guidelines, including the optimal approach to iron overload evaluation and monitoring as well as toxicity monitoring during chelation treatment. Early initiation of chelation is highly recommended. First line therapy is deferoxamine or deferasirox, dependent on patient profile and preferences. Chelation is initiated in view of evidence of iron overload (ferritin >500 ng/mL, transferrin saturation >60%, or elevated liver iron content on MRI). Reduced dosing should be considered in children under labeled age limits (age 3 years for deferoxamine and 2 years for deferasirox). The therapy must be frequently adjusted on the ground of efficacy and toxicity (23).

#### 3.2.2 Developmental interventions

##### 3.2.2.1 Leucine

Parallel to pre-clinical models (77–79), the amino acid leucine has been shown to induce erythroid response and improved growth in some patients with DBA syndrome, this was associated with very good safety profile (NCT01362595) (80, 81). Further studies with leucine for steroid-responsive DBA syndrome are open and some centers use leucine in selected DBA patients (82).

##### 3.2.2.2 Eltrombopag

So far reported response rate to eltrombopag in DBA are relatively low, although there are some responders. Perhaps, eltrombopag could be more beneficial in patients with particular genetic variants (83).

### 3.3 Fanconi anemia

#### 3.3.1 Established interventions

##### 3.3.1.1 Androgens

Oxymetholone and danazol were reported to result in a transient or relatively prolonged hematologic response in majority of patients within 3 months of treatment, as a therapeutic option in patients who are at the moment not eligible for HSCT (11, 20, 84). The reports on relative response rates for different androgens (especially oxymetholone versus danazol) are conflicting. Despite well documented, dose-related, side effects the advantage of androgens include long history of their use and the absence of therapy-related mortality. While, some initially responsive patient will become refractory over time, it appears that up to 20% of patients receiving continuous low dose androgens might never need HSCT. Androgens do not lower the risk of clonal progression. The improved transplant procedures and the advent of other therapies, including gene therapies, may further limit the use of androgens in FA but there are still clinical scenarios, such as lack of transplant eligibility or consent, in which the use of androgens will be beneficial, based on individual decisions (20, 84). Dosing, timeframes and practical considerations of androgen administration in FA are discussed in recent recommendations (84).

##### 3.3.1.2 G-CSF

G-CSF might be considered in FA patients with severe neutropenia associated with or serious infections as a salvage therapy or bridge to transplantation. A starting dose of around 5 µg/kg/day I recommended with the target of absolute neutrophil count (ANC) of greater than 1,000/mm^3^. The dose should be tapered to the lowest effective dose, perhaps with 2–3 injections weekly (32).

#### 3.3.2 Developmental interventions

##### 3.3.2.1 Metformin

The evidence for metformin clinical potential in FA is derived from a single trial at Boston Children’s Hospital of metformin in nondiabetic patients with FA aiming to determine feasibility and tolerability of metformin treatment and to assess for improvement in blood counts (NCT03398824). Twelve of patients completed 6 months of metformin treatment. No grade 3 or higher adverse events potentially attributed to metformin were reported. Four patients (30.8%) achieved hematological response on metformin therapy according to predefined Myelodysplastic Syndrome International Working Group criteria. The interpretation of the results is difficult and a fluctuating manner of hematological values in FA must be acknowledged. Nonetheless, there is some potential for metformin slowing progression of bone marrow failure and also as a preventive agent aimed at diminishing cancer risk in FA (56, 57).

##### 3.3.2.2 Quercetin

A single-center, prospective, phase 1 dose-finding study (with an expansion cohort) of quercetin for patients with FA was recently reported (identifier: NCT01720147) from Children’s Hospital Medical Center, Cincinnati. Quercetin was well tolerated. The authors claim that 8 of 15 patients (53%) with cytopenia had a hematological response at some point following quercetin therapy based on similar criteria as in the above mentioned FA metformin study. The interpretation is difficult due to a degree of fluctuation of hematological values in FA patients. At the moment, the potential for clinical application of quercetin for marrow failure in FA is very limited. On the other hand, the authors demonstrated significant drop in ROS levels in peripheral blood and bone marrow of FA patients during treatment, suggesting quercetin could for example exert long-term cancer-preventing effects (85). Consistently, Quercetin Chemoprevention for Squamous Cell Carcinoma in Patients With Fanconi Anaemia trial is active (NCT03476330).

##### 3.3.2.3 Eltrombopag

There are a few reports of successful use of eltrombopag in specific FA patients. Those included bridging to HSCT scenario, in a mosaic FA patient and in two FA patients who previously received gene therapy infused with low numbers of gene-corrected HSCs. On the other hand FANCREV study (NCT06045052), did not demonstrate clinically relevant responses to eltrombopag in FA patients, apart from mosaic and gene-therapy patients. The clinical use of eltrombopag in FA remains this very limited (86, 87). There are no data for romiplostim efficacy either, with just one report of post-transplant use.

##### 3.3.2.4 Ataluren and amlexenox

There is also single-report of the effects of a combination of ataluren and an anti-inflammatory drug amlexenox in an FA model. The authors believe the results could indicate a cancer-prevention potential for that combination (55).

### 3.4 Telomere-biology disorders/dyskeratosis congenita

#### 3.4.1 Established interventions

##### 3.4.1.1 Androgens

Androgen derivatives have been in use in bone marrow failure since 1960s. Several studies reported hematological responses upon androgen in dyskeratosis congenita patients. The most frequently used agent is danazol at a mean adult daily dose of 600–800 mg (28, 29). The other active androgen derivatives are nandrolone decanoate (5 mg/kg every two weeks) and oxymetholone (at an initial dosage of 0.5–1 mg/kg/day), generally, patients respond to treatment within the first 6 months. There is a lack of studies directly comparing efficacy and toxicities of different agents. There is some evidence for relatively lowest virilization and hepatotoxicity profile for danazol (19, 20, 28). The duration of treatment is not well established and varies among studies. Prolonged treatment is especially problematic in children because of the risk of virilization and growth failure (28). Dose tapering is recommended but the evidence for that is low. Importantly, there is also some limited evidence that androgens might improve clinical course of telomeropathy-related interstitial lung disease (ILD). Also quality of life of ILD patients was reported to be improved, potentially partly due to higher hemoglobin levels (28).

In summary, androgen treatment remains important therapeutic option in telomere-biology disorders. This area was recently extensively reviewed by Vieri et al (28).

#### 3.4.2 Developmental interventions

##### 3.4.2.1 Poly(A) polymerase PAPD5 inhibitors

Small-molecule PAPD5 inhibitors were reported to induce telomere restoration *in vitro* in stem cell models of cell derived from DC patients (65, 66, 88, 89). PAPD5 inhibitor, RG-7834, is in development as an oral HBV therapeutic belonging to the dihydroquinolidinone compound family, functioning in that setting as a HBV RNA destabilizer (90). We are not aware of any on-going clinical trials on PAPD5 inhibitors in telomere-biology disorders.

### 3.5 Shwachman-Diamond syndrome

#### 3.5.1 Established interventions

##### 3.5.1.1 Pancreatic enzymes, vitamins

One of the major, but variable, clinical features of SDS is exocrine pancreatic dysfunction that might be associated with nutrient maldigestion. Electrolyte and fluid secretion in the ducts remains normal, but the secretion of proteolytic enzymes is severely compromised resulting in steatorrhea and reduced levels of fat-soluble vitamins (A, D, E, K). Exocrine pancreatic function may gradually improve in childhood. Consequently, the pancreatic function of SDS patients should be occasionally reassessed, to guide the need for supplementation. In general, if steatorrhea is evident pancreatic enzyme replacement should be implemented. The initial dose is 2,000 lipase units/kg body weight daily. The dosing generally follows the guidelines for cystic fibrosis (maximum 10,000 lipase units/kg body weight daily). Pancreatic enzymes should be taken with all meals that contain protein, fat or complex carbohydrates. Proton pump inhibitor may be also considered. Blood levels of fat-soluble vitamins should be measured 1–2 times per year, especially in young children, and supplementation should be considered with low levels. Vitamin K may be administered in a rare case of a need of correction of prolonged prothrombin time (2).

##### 3.5.1.2 G-CSF

G-CSF in patients with SDS is recommended for a limited period and in case of severe or recurrent infections. This is due to intrinsic high risk of myeloid neoplasia is SDS. Strict hematologic surveillance of SDS patients on G-CSF is required, and the minimal effective dose of G‐CSF is preferred (1–4 µg/kg/d). The starting dose is 1 µg/kg/d recommended (42, 43).

#### 3.5.2 Developmental interventions

##### 3.5.2.1 Ataluren

In a recent compassionate programme ataluren treatment of 3 patients over 12 months resulted in a rise in SBDS protein levels, improved myelopoiesis markers, improved absolute neutrophil count in two out of three patients, and improved platelet count in all 3 recruited patients (54, 91, 92). Nevertheless, also taking into account marketing authorization issues, the impact of ataluren on SBDS management is and will likely remain minimal.

### 3.6 Severe congenital neutropenia

#### 3.6.1 Established interventions

##### 3.6.1.1 G-CSF

The data from large neutropenia registries demonstrated that G-CSF treatment of patients with SCN (including CyN) results in survival benefit, lower rate of infections and infectious complications, hospital stays, and improved quality of life as compared to historical data. Currently, the threshold of ANC of 1.0 × 10^9^/L is considered appropriate to protect against infections and is a target for G-CSF dosing in SCN. Patients with cyclic neutropenia are usually effectively treated with G‐CSF, at doses of 1–5 µg/kg/d (starting dose <3 µg/kg/d). Patients with SCN receive a starting dose of 5 µg/kg/d. Some may require higher dose but that is associated with increased risk of progression to MDS/AML and a need for close surveillance for myeloid neoplasm (41–43). This is presented with more detail, including the potential us of PEG-filgrastime in selected patients, in recent guidelines (41, 42).

##### 3.6.1.2 SGLT2 inhibitors in GSD type 1b and G6PC3-deficiency

Very satisfactory impact on neutrophil count and function, as well as infection rate, was recently described in 7 patients with GSD1b of empagliflozin as compared with the reference treatment with granulocyte-colony stimulating factor (G-CSF). G-CSF injections was discontinued in 6 patients without deterioration. This, has demonstrated the superior efficacy of empagliflozin compared with G-CSF (EudraCT 2021-000580-78.) (59).

Clinical studies of SGLT2 inhibitors in expanded groups of children with GSD1b and/or G6PC3 are currently in progress, but the results are not yet available (ClinicalTrials.gov Identifiers: NCT04986735, NCT04138251, NCT05078879).

##### 3.6.1.3 CXCR4 antagonists in WHIM syndrome (plerixafor, mavorixafor)

Following preclinical studies, plerixafor was shown in 2010s to improve hematological parameters, infection rates and quality of life of WHIM syndrome in several small studies of limited number of patients and in a phase 1 clinical trial (47). An important study of 3 adults with WHIM syndrome with severe syndromes and comorbidities who could not qualify for clinical trials by McDermott et all reported improvement in all hematological lineages and wart burden, lower frequency of infections and stabilization of HPV–associated oropharyngeal squamous-cell carcinoma (48).

More recently, a phase III randomized crossover trial of plerixafor versus G-CSF for treatment of WHIM syndrome demonstrated plerixafor demonstrated non-inferiority to G-CSF for maintaining neutrophil counts of more than 500 cells/μL and superiority to G-CSF for maintaining lymphocyte counts above 1,000 cells/μL. Regression of large warts occurred in 5 out of 7 plerixafor patients. Quality of life and adverse events were comparable (93).

Daily, oral mavorixafor was demonstrated in a blinded, placebo-controlled, phase 3 trial (#NCT03995108) to increase lymphocyte and neutrophil counts and reduce infection load, with minimal side effects (49).

In conclusion, CXCR4 antagonists are well tolerated, clinically effective agents in WHIM syndrome. Their role in WHIM syndrome management versus (or parallelly to) G-CSF, immunoglobulin replacement therapy and HSCT must be based on patients medical profile and preferences. The oral route of administration for mavorixafor is highly advantageous in that context.

Somewhat surprisingly, mavorixafor is tested in a phase 2 study for chronic neutropenia ‘non-WHIM’ patients (NCT04154488) with or without concurrent G-CSF. Preliminary report of three ‘non-WHIM’ chronic neutropenia patients demonstrated durable rise in ANC compared with baseline in 3 study participants. G-CSF tapering was implemented in 2 study participants following ANC increase after the first 2 months of treatment, NCT04154488 (94). The complete results of the study are not yet available, but the interim analysis results as presented by the manufacturer are promising and further studies are scheduled.

### 3.7 Inherited thrombocytopenias

#### 3.7.1 Thrombopoietin-receptor agonists

There is some evidence for the potential clinical use of thrombopoietin receptor agonists in IT. However, published evidence so far has been largely limited to small, non-randomized studies, which is not surprising considering the low frequency of the disorders (95).

The first encouraging report was published by Pecci et al. in 2010 and described short-term eltrombopag use in the most common form of IT worldwide caused by MYH9 mutations. Of the 12 study participants, 11 patients experienced an increase in platelet counts, in most cases exceeding 100 ×10^9^/L, without significant adverse events (96). Further reports supported the use of TPO-RAs as an alternative to platelet transfusions before hemostatic challenges in IT and as bridging therapy to HSCT in Wiskott–Aldrich syndrome (95, 97–99). In 2020, a phase II study reported outcomes of long-term eltrombopag therapy in 24 patients with various subtypes of IT, with only two non-responders (100). Not surprisingly, the most striking results were observed following the administration of romiplostim in patients with congenital amegakaryocytic thrombocytopenia (CAMT) caused by THPO mutations. In this rare disorder treatment with TPO-RA results in improvements across all hematopoietic lineages (101). Overall, TPO-RAs appear to play an increasingly important role in the management of IT. Nevertheless, long-term follow-up data needs to be acquired, particularly in patients with cancer-predisposition syndromes, such as ETV6-, RUNX1-, and ANKRD26-related thrombocytopenia.

### 3.8 Practical considerations for monitoring of major classes of medical therapies in IBMFs

This is summarized and presented in **Table 2**.

**Table 2:** IBMF-focused risk and practical pharmacotherapy monitoring recommendations.

Therapy	IBMFS-specific risks	Practical monitoring
Androgens (danazol, oxymetholone, nandrolone)	Virilization/endocrine effects, hypertension, cholestasis and hepatic tumors.	Before starting: document baseline blood counts, liver tests (including a cholestasis profile), and blood pressure, with lipids and a brief puberty/growth assessment when relevant, consider baseline liver imaging. During treatment: monitor liver tests, blood pressure, and virilization/acne or behavioral changes, growth velocity and bone age in children. Monitor for hepatic lesions.
G-CSF (filgrastim, pegfilgrastim)	May mask progression of marrow failure. In SDS, due to intrinsic high myeloid neoplasia risk use minimal effective dose, strict surveillance.	During treatment: titrate G-CSF to the lowest dose that maintains an infection-protective ANC, and plan periodic marrow surveillance. Escalate evaluation if the dose requirement rises, anemia or thrombocytopenia appears, atypical cells/blasts are seen, response is lost, or splenomegaly develops.
TPO-receptor agonists (eltrombopag, romiplostim)	In cancer-predisposition thrombocytopenias (ETV6/RUNX1/ANKRD26) possible acceleration of CH, and MDS/AML. Hepatopathy. Bone marrow fibrosis.	During treatment: monitor for excessive platelet rises, liver tests, and thrombotic symptoms. New cytopenia, atypical cells/blasts are consideration of bone marrow evaluation, any thrombotic event, or liver test elevation should prompt reassessment of risk–benefit.
SGLT2 inhibitors (empagliflozin) in GSD1b/G6PC3 deficiency	Metabolic, and renal complications.	During treatment: monitor genital/urinary infection frequency and renal function tests
Iron chelation (deferasirox, deferoxamine) in transfusion-dependent IBMFS	Renal function impairment.	During treatment: monitor iron overload symptoms with ferritin trends (with liver/heart MRI where available) and document renal and hepatic function. Assess hearing/vision when relevant for the chosen chelator. Escalate evaluation if creatinine rises or proteinuria appears, transaminases remain persistently elevated, or the patient develops symptoms of auditory/visual toxicity.

## 4 Discussion and future directions

Inherited bone marrow failure syndromes (IBMFs) are associated with reduced expected life expectancy, hematopoietic failure and disease-specific as well as non-specific infection-related organ sequelae.

Non-cellular medicines used as bridging may improve blood counts and diminish the occurrence of complications, nevertheless, they can also mask the early onset of marrow failure, increase selection pressure in hematopoiesis, and complicate the timing of HSCT. For example, in the context of SDS, G-CSF is advised to be applied cautiously due to the inherent risk of myeloid neoplasia, accompanied by a recommendation for hematologic monitoring and the administration of the minimal effective dosage.

At the moment the only definitely curative approach to improve failing hematopoiesis in allogeneic hematopoietic stem cell transplantation. This remains associated with significant transplant-related mortality and morbidity, including in particular GvHD and increased risk of solid cancers.

Some patients are not eligible for transplant or risk assessment does not justify HSCT (3, 7–9). Even in view of development of gene therapy and genome correction technologies, there remains a wide range of applications for medical therapies in IBMFs. These range from nearly curative steroids in DBA or androgens providing lasting hematological responses in DC, to developmental reactive oxygen species and aldehyde scavengers in FA, and pre-clinical approaches for telomere elongation in DC. Also there remains a need for amelioration of non-hematopoietic phenotypes and morbidities in IBMFs, such as recently achieved improvement of cardiac function with elamipretide in Barth syndrome patients leading to accelerated FDA approval in that indication on September 19,2025. In general, in view of the improved understanding of the molecular mechanisms of IBMFs, the progress so far has been very disappointing and the clinically important interventions were introduced based on empirical experience several decades ago (3, 7). Currently, the researchers and practitioners are increasingly aware of the disease-specific molecular mechanism leading the disease phenotype in IBMFs, which should eventually lead to a new are of pathogenesis-driven interventions.

A particularly valid example where such mechanism-driven interventions could be envisioned is the setting of SDS. Most SDS cases are due to pathogenic variants in SBDS, which cooperates with EFL1 (also mutate in SDS) to release eIF6 from pre-60S to facilitate assembly of mature 80S ribosome. eIF6 is an anti-association factor that blocks the joining of the 40S and 60S ribosomal subunits and thus prevents 80S that is necessary for activating translation. This leads to down-stream abnormalities including increased apoptosis and TP53 activation. EIF6 is frequently somatically mutated in tissues of SDS patients, and such loss-of-function mutations lead to lower content and lower inhibitory capacity of eIF6 on pre-60S, resulting in improved 80S maturation, translation and amelioration of the disease phenotype (102–104). This is a fascinating example of natural ‘gene therapy’ suggesting that any strategy at removing eIF6 from the complex or otherwise increasing 80S maturation could be therapeutic in SDS. While eIF6 is currently undruggable, many cellular proteins are or will become in the future targetable for example via ‘molecular glue degraders’ or other technologies. This is facilitated by the concept of drug repurposing (105). Apart from that, basic knowledge of 80S assembly provides other, perhaps easier targets, whose inhibition/degradation/knock-down should provide similar consequences as eIF6 loss. These potential targets include for example ZNF622 and HECTD1. ZNF622 is a 60S assembly factor, whose accumulation prevents release of eIF6 from 60S and ribosome maturation. ZNF622 loss/knock-down might be predicted to lead to similar consequences for SDS-cells as eIF6 loss. Indeed, ZNF622 is in physiological hematopoiesis degraded HectD1 ubiquitinase and in experimental setting HectD1 loss leads to 60S/40S joining defects and impaired hematopoietic regeneration (102, 104, 106, 107).

This is just an example of pathways that could be targeted, some supported by the effects of somatic mutations leading to phenotypic correction/reversion described in patients. Similar mechanism-driven approaches could be envisioned in other IBMFs. Medical therapies will continue to be especially important for tissues other than hematopoietic, that cannot be replaced or corrected with HSCT or autologous gene-corrected transplant, including in particular solid cancer prevention interventions.

## 5 Conclusions

The significance of non-cellular therapies in IBMFs varies from nearly curative steroid therapy in DBA to minimal role in several other syndromes. Generally the impact of medical interventions in management IBMFs is likely to be diminishing due to the advent of gene and genome-correction approaches. On the other hand, there is an accumulating knowledge on IBMFs pathogenic mechanisms and this may lead to new era of low-toxicity molecularly targeted therapeutics, likely benefiting from the concept of drug repurposing.

Further, some medical interventions will likely remain important in controlling extra-hematopoietic signs and complications, and in particular solid cancer prevention interventions. In IBMFs, the safety signs to monitor include not only adverse events but also time-dependent trajectories, such as clonal dynamics, cancer development and cumulative organ injury.
